# Mutation spectrum of TP53 gene predicts clinicopathological features and survival of gastric cancer

**DOI:** 10.18632/oncotarget.9770

**Published:** 2016-06-01

**Authors:** Tomomitsu Tahara, Tomoyuki Shibata, Yasuyuki Okamoto, Jumpei Yamazaki, Tomohiko Kawamura, Noriyuki Horiguchi, Masaaki Okubo, Naoko Nakano, Takamitsu Ishizuka, Mitsuo Nagasaka, Yoshihito Nakagawa, Naoki Ohmiya

**Affiliations:** ^1^ Department of Gastroenterology, Fujita Health University School of Medicine, Toyoake, Japan; ^2^ Department of Gastroenterology and Metabolism, Nagoya City University Graduate School of Medical Sciences, Nagoya, Japan; ^3^ Laboratory of Molecular Medicine, Hokkaido University Graduate School of Veterinary Medicine, Sapporo, Japan

**Keywords:** TP53 mutation, gastric cancer, spectrum, survival, hotspot mutations

## Abstract

**Background and aim:**

*TP53* gene is frequently mutated in gastric cancer (GC), but the relationship with clinicopathological features and prognosis is conflicting. Here, we screened *TP53* mutation spectrum of 214 GC patients in relation to their clinicopathological features and prognosis.

**Results:**

*TP53* nonsilent mutations were detected in 80 cases (37.4%), being frequently occurred as C:G to T:A single nucleotide transitions at 5′-CpG-3′ sites. *TP53* mutations occurred more frequently in differentiated histologic type than in undifferentiated type in the early stage (48.6% vs. 7%, *P*=0.0006), while the mutations correlated with venous invasion among advanced stage (47.7% *vs.* 20.7%, *P*=0.04). Subset of GC with *TP53* hot spot mutations (R175, G245, R248, R273, R282) presented significantly worse overall survival and recurrence free survival compared to others (both *P*=0.001).

**Methods:**

Matched biopsies from GC and adjacent tissues from 214 patients were used for the experiment. All coding regions of *TP53* gene (exon2 to exon11) were examined using Sanger sequencing.

**Conclusion:**

Our data suggest that GC with *TP53* mutations seems to develop as differentiated histologic type and show aggressive biological behavior such as venous invasion. Moreover, our data emphasizes the importance of discriminating *TP53* hot spot mutations (R175, G245, R248, R273, R282) to predict worse overall survival and recurrence free survival of GC patients.

## INTRODUCTION

Gastric cancer (GC) is one of the most common malignancies worldwide, accounted for approximately 70,000 new cases and 650,000 deaths per year [1, 2]. Despite advances in diagnosis technologies, many patients still have advanced disease at diagnosis and treatment outcomes for such patients are poor [3]. Moreover, GC is heterogeneous in its clinical course and prognosis. However, underlying mechanisms to address this issue has not been fully understood.

Concerning the molecular abnormality in GC, several genetic changes in gastric carcinogenesis have been elucidated including oncogenes (*KRAS*, *b-catenin*, *ERBB2*, *PIK3CA* etc.) [4–7] and tumor-suppressor genes (*CDH1*, *p16*, *TP53*, *ARID1A* etc.) [8–11]. *TP53* is the most studied gene in many cancers and may be possible candidate biomarker for GC. The gene encoding *TP53* is located on chromosome 17p and consists of 11 exons and 10 introns. TP53 protein has important cellular functions, including in cell cycle regulation, apoptosis, and DNA repair [12, 13]. Even by advances in new technologies, such as whole exome sequencing, *TP53* is confirmed as the most frequently mutated gene in human cancers, with alterations occurring in about half of human cancers [8–11].

Concerning the association between *TP53* status and subtypes and prognosis of GC, conflicting results did not conclude the clinical significance of *TP53* as a molecular marker [14–17]. We reasoned that conflicting result may be partly due to the limitation of immunohistochemistry assay that may not detect truncating type mutations (frameshift and nonsense mutations) [14], or a lack of discriminations of different types of mutation occurring throughout the coding exons. To resolve these issues, we investigated *TP53* mutation spectrum throughout the entire exons in 214 GC cases. We aimed to clarify the association between clinicopathological features and prognosis of GC and *TP53* mutation status with an attempt to their change types.

## RESULTS

### Spectrum of TP53 mutations

**Figure 1:**
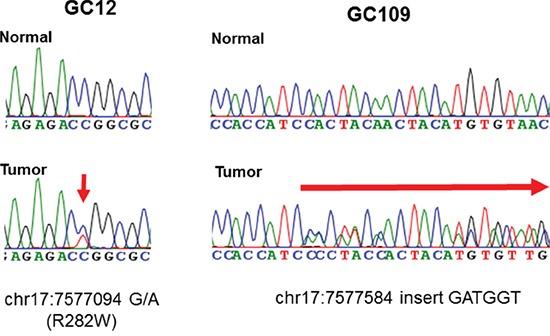
Representative sequencing chromatograms of two cases with *TP53* mutations Below indicates the position at chromosome. Position at codon was indicated in the parentheses (left).

We performed Sanger sequencing of *TP53* genes in 214 GC tissues and matched normal tissues Representative sequencing chromatograms are shown in Figure 1.

**Figure 2:**
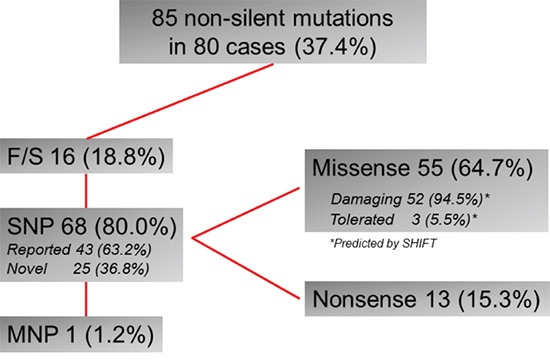
Summary of *TP53* mutations in 214 GC cases F/S, frame shift; SNP, single nucleotide polymorphism; MNP multiple nucleotide polymorphism;

**Figure 3:**
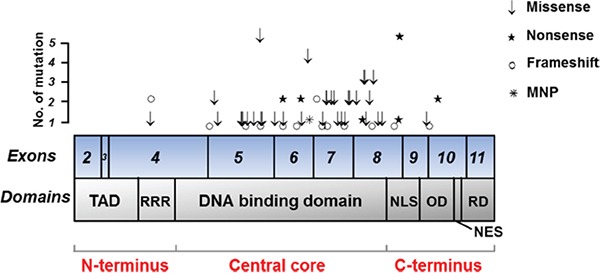
Mutation spectrum of *TP53* in 214 GC cases Individual exons are represented as numbered boxes. TAD, Trans activation domain; RRR, Proline-rich region; NLS, nuclear localization signal sequence; OD, Tetramerization domain; NES, nuclear export signal sequence; RD, Regulatory domain;

We found 80 cases (37.4%) with *TP53* nonsilent mutations. One tumor had 3 mutations, 3 had 2 mutations, and 76 had a single mutation, for a total of 85 mutations in this cohort. Mostly, these mutations were predicted to either truncate the protein through base substitutions, resulting in a stop codon (nonsense, 13 mutations) or a frame shift (16 mutations), multiple nucleotide polymorphism (1 mutation) or to damage the protein as predicted by SIFT analysis (52 out of 55 missense mutations) (Figure 2, Supplementary Table 2). These mutations were distributed across exon4 to exon10, but majority were in the DNA binding domain of central core region (Figure 3). Concerning the spectrum and nucleotide contexts of the substitution mutations, most frequent alterations were C:G to T:A transitions (54.4%), predominantly occurred at 5′-CpG-3′dinucleotide sites (47.1%, Table 1).

**Table 1:** Spectrum of *TP53* single base substitutions

Variables	n (%)
*Total number of substitutions*	68
*Substitutions at C:G base pairs*	
C:G → T:A	37 (54.4)
C:G → G:C	3 (4.4)
C:G → A:T	8 (11.7)
*Substitutions at T:A base pairs*	
T:A → C:G	9 (13.2)
T:A → G:C	3 (4.4)
T:A → A:T	6 (8.8)
*Substitutions at specific dinucleotides$*	
5′-CpG-3′	32 (47.1)
5′-TpC-3′	12 (17.6)

Base substitutions in coding sequences resulting in nonsynonymous changes are included (see Figure 2).

$ Includes substitutions at the C or G of the 5′-CpG-3′ dinucleotide, the C of the 5′-TpC-3′ dinucleotide, or the G of the 5′-GpA-3′ dinucleotide.

### Association between TP53 mutations and clinicopathological subtypes of GC

We investigated whether *TP53* mutation positivity is associated with any clinicopathological subtypes of GC. Age, gender, location, *H. pylori* status, staging, histology, venous and lymphatic invasion, lymph node, liver, peritoneal and other distant metastasis were included for this analysis. Although we did not find any association between *TP53* mutation positivity and age, gender, location, *H. pylori* status and staging (Table 2), *TP53* mutation was more frequent in differentiated histologic type, compared to undifferentiated type (44.0% *vs.* 26.9%, *P*=0.01). Notably, this association was more striking among early stage GC (T1, 48.6% vs. 7%, *P*=0.0006) but not in advanced stage (T2<, 35.0% vs. 36.4%, *P*>0.1) (Table 3). We also found that in advanced stage GC (T2<), *TP53* mutation was more frequent in cases having venous invasion (47.7% *vs.* 20.7%, *P*=0.04). A similar trend was also found in cases having more severe lymph node metastasis (N0~1 *vs.* N2~, 31.3% vs. 47.1%, *P*<0.1) (Table 4).

**Table 2:** *TP53* mutation status and subtypes of GC

Variables n (%)	*TP53* wild type	*TP53* mutated
*Age (mean +/− SEM)*	64.4+/−1.0	66.4+/−1.4
*Gender*		
Female n (%)	46 (67.6)	22 (32.4)
Male n (%)	88 (60.3)	58 (39.7)
*Location*		
Cardia n (%)	7 (70.0)	3 (30.0)
Upper n (%)	19 (65.5)	10 (34.5)
Middle n (%)	70 (64.8)	38 (35.2)
Lower n (%)	38 (56.7)	29 (43.3)
*H. pylori status*		
Negative n (%)	19 (65.5)	10 (34.5)
Positive n (%)	115 (62.2)	70 (37.8)
*Stage*		
I n (%)	70 (66.0)	36 (34.0)
II n (%)	20 (64.5)	11 (35.5)
III n (%)	21 (58.3)	15 (41.7)
IV n (%)	22 (55.0)	18 (45.0)
*Depth*		
T1 n (%)	62 (63.3)	36 (36.7)
T2 n (%)	16 (66.7)	8 (33.3)
T3 n (%)	8 (80.0)	2 (20.0)
T4 n (%)	48 (58.5)	34 (41.5)

Stage was determined for 213 cases

**Table 3:** *TP53* mutation status and histologic subtypes of GC

Variables n (%)	*TP53* wild type	*TP53* mutated
*Total number of patients*	134 (62.6)	80 (37.4)
*All subjects (n=214)*#		
Differentiated n (%)	61 (56.0)	48 (44.0)
Un differentiated n (%)	68 (73.1)	25 (26.9)
Mixed n (%)	5 (41.7)	7 (58.3)
*Early stage (T1, n=98)*$		
Differentiated n (%)	35 (51.5)	33 (48.5)
Un differentiated n (%)	26 (92.9)	2 (7.1)
Mixed n (%)	1 (50.0)	1 (50.0)
*Advanced stage (T2~4, n=116)*		
Differentiated n (%)	26 (65.0)	14 (35.0)
Un differentiated n (%)	42 (63.6)	24 (36.4)
Mixed n (%)	4 (40.0)	6 (60.0)

# *P*=0.01;

$ *P*=0.0006;

**Table 4:** *TP53* mutation status and lymphatic and venal invasion, and metastasis in advanced GC

	*TP53* wild type	*TP53* mutated
*Lymphatic invasion*		
Negative	4 (80.0)	1 (20.0)
Positive	42 (61.8)	26 (38.2)
*Venous invasion*#		
Negative	23 (79.3)	6 (20.7)
Positive	23 (52.3)	21 (47.7)
*Lymphnode metastasis*$		
N0~1	44 (68.8)	20 (31.3)
N2~	27 (52.9)	24 (47.1)
*Peritoneal dissemination*		
Negative	56 (65.9)	29 (34.1)
Positive	15 (50.0)	15 (50.0)
*Liver metastasis*		
Negative	66 (61.1)	42 (38.9)
Positive	5 (71.4)	2 (28.6)
*Other distant metastasis*		
Negative	66 (61.7)	41 (38.3)
Positive	5 (62.5)	3 (37.5)

Lymphatic and venous invasion was determined for 73 cases

Metastasis was determined for 115 cases

# *P*=0.04;

$ *P*<0.10;

### Association between TP53 hot spot mutations and clinicopathological subtypes of GC

**Figure 4:**
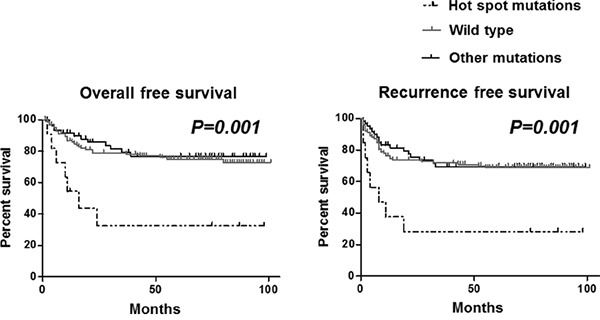
Association between *TP53* mutation and overall survival (left) and recurrence free survival (right) in GC patients Statistical analysis was performed by the Log rank test.

Next we aimed to investigate whether *TP53* mutation is associated with survival and relapse of GC patients. 210 cases were evaluated for this analysis. We did not find any significant association between overall survival and recurrence free survival among cases with or without *TP53* mutation (data not shown). However, focusing on *TP53* mutation hot spots within the central core (R175, G245, R248, R273, R282), which is reported to be mutated across various cancers. We found that the cases having mutation in this hot spots (6.2%, n=13) presented significantly worse overall survival and recurrence free survival compared to others (both P values =0.001, Figure 4). To confirm this result, we next conducted multivariate survival analysis using Cox's regression model to see whether the *TP53* hotspot mutations would be associated with worse overall survival as an independent factor. Age, gender, histology, depth, lymph node metastasis, peritoneal dissemination, liver or other distant metastasis, staging and *TP53* hotspot mutations were included for this analysis. The result demonstrated that *TP53* hotspot mutations was associated with worse overall survival as an independent factor (hazard ratio=3.57, 95% confidence interval= 1.44-8.86, P=0.006, Table 5).

**Table 5:** Multivariate survival analysis using Cox's regression model for adjustment of clinicopathological factors *TP53* hotspot mutations

Variables	HR (95%CI)	*P*
Age	1.02 (1.00-1.05)	0.05
Gender (male)	1.74 (0.88-3.16)	0.11
*H. pylori* positive	0.81 (0.32-2.08)	0.66
Location (cardia)	3.61 (1.22-10.65)	0.02
Histology (un differentiated type)	1.11 (0.57-2.17)	0.11
Depth	2.12 (1.08-4.13)	0.03
Lymph node metastasis	1.69 (0.72-3.95)	0.23
Peritoneal dissemination	2.86 (0.81-10.1)	0.1
Liver metastasis	1.92 (0.36-10.37)	0.45
Other distant metastasis	1.45 (0.45-4.65)	0.53
Staging	1.77 (0.64-4.84)	0.27
*TP53* hotspot mutations	3.57 (1.44-8.86)	0.006

HR, hazard ratio; CI, confidence interval;

We also tried to investigate the association between *TP53* hotspot mutations and recurrences of GC including liver, peritoneal, lymph node and others. In advanced GC (T2<), this analysis showed that the cases that have *TP53* hotspot mutations presented significantly higher liver or other recurrences compared to others (*P*<0.05, 0.02, respectively) (Table 6).

**Table 6:** Association between *TP53* mutation in hot spots and recurrence in advanced GC

Variables	*TP53* hot spot mutations	Others
	(n=11)	(n=105)
Over all recurrences n (%)	7 (63.6)	47 (44.7)
Liver recurrences n (%)#	3 (27.3)	8 (7.6)
Peritoneal recurrences n (%)	3 (27.3)	32 (30.5)
Lymph node recurrences n (%)	1 (9.1)	6 (5.7)
Other recurrences n (%)$	3 (27.3)	6 (5.7)

# *P*<0.05;

$ *P*=0.02;

We also evaluated whether the change types (nonsense, missense or frame shift), nucleotide contexts of the substitution mutations, and locations (exons, 5′-CpG-3′ sites 5′-TpC-3′) of *TP53* mutations would be associated with prognosis of GC. But association were all negative except for the mutations in the hot spots (data not shown).

## DISCUSSION

Regarding the spectrum of *TP53* mutations in our study, C:G to T:A transitions were predominantly occurred at 5′-CpG-3′dinucleotide sites, which was in line with other study in GC [8, 10, 14]. Not only the *TP53* gene, C:G to T:A transitions are known to be most frequent transitions throughout the genomes in GC [8, 10]. C to T transition is induced by nitric oxide [19, 20], a substance known to be induced by *H. pylori* infections. C:G to T:A transitions are also specifically induced by N-methyl-N'-nitro-N-nitrosoquanidine and N-nitroso compounds found in foods, substances considered to be carcinogens in GC [21]. These foods are commonly consumed in high risk populations for developing GC including Japanese. Our result confirmed that C:G to T:A transitions are typical mutation spectrum of *TP53* in GC, reflecting interaction between *H. pylori* infection and environmental predisposing factors.

There are conflicting results with respect to the prevalence of *TP53* mutations as well as their relationship to clinicopathological features in GC [14], which may be partly due to difference of patient constitutions or the method for detection of mutations. Immunohistochemistry assay used in many studies lacks the ability to detect truncating type mutations (frameshift and nonsense mutations). Single strand conformation polymorphism (SSCP) analysis also misses considerable percentage of mutations [14]. In this study, all coding exons of *TP53* gene were screened by the Sanger sequencing. *TP53* mutation frequency in our dataset was 37.4%, which was quite similar to that of recent Japanese study using the Digital PCR targeted sequencing coupled with macrodissection [11]. Other strength of our study was to use endoscopic biopsy specimens. Since the mutation detection rate seemed to be reliable even by using tiny biopsy samples, our data suggests the potential usefulness of endoscopic biopsy for the molecular analysis especially in patients with advanced stage who have no information from surgically resected specimens.

Among several clinicopathological features, *TP53* mutation was more frequent in differentiated histologic type, which is in line with some previous reports [22, 23]. Of note, the correlation was striking among early stage, not observed in advanced stage. The data suggests that majority of *TP53* mutated GC are differentiated histologic type in the early stage. It has been reported that progression of differentiated type GC often results in histological change to undifferentiated type [24]. It is therefore possible that considerable portion of *TP53* mutated undifferentiated GCs may have originally developed as differentiated type. We also found that *TP53* mutation was correlated with venous invasion in advanced GC. A similar trend was also found for more severe lymph node metastasis. This indicates mutation status reflects different biological behavior of GC. *TP53* mutation has also been associated with more aggressive phenotypes of other tumor types, such as esophageal and colorectal cancers [25, 26]. Since GC with venous invasion and severe lymph node metastasis often show more aggressive biological behaviors including relapsing even after curative surgery, *TP53* mutated GC may need more intensive adjuvant chemotherapy or frequent follow up to prevent relapsing after gastrectomy.

We have also shown that subset of GCs with specific *TP53* mutations (hot spots within the central core: R175, G245, R248, R273, R282) is associated with worse overall survival and recurrence free survival. The worse prognosis of these mutations was also confirmed as an independent factor using the multivariate survival analysis. The same mutation type was also associated with recurrences of GC. This result emphasizes the importance of discriminating *TP53* mutations to divide GC patients for better clinical implementation reflecting biological behavior. Our data suggest that hotspot *TP53* mutated GC may need more intensive therapy or careful follow up even after curative resection. Importance of discriminating *TP53* change types is also reported in colorectal cancer patients with poor prognosis who have mutations in R175 and G245. Although the prevalence of these mutations are not so high in all GC in our dataset (13/214, 6.2%). Significant worse prognosis of these patients suggests the potential usefulness of these mutations as a molecular marker in GC patients. Since the relationship between *TP53* status and response to chemotherapy in GC patients has been conflicting [15–17], whether these mutations can predict response to chemotherapy needs to be evaluated in the larger cohort. We analyzed *TP53* mutations in GC tissue, obtained by endoscopic biopsy. It should be noted that the results may not reflect the characteristics of whole part of the tumor considering the tumor heterogeneity. Still, our result showing the association between *TP53* hotspot mutations and prognosis of GC would provide considerable information especially in patients who cannot undergo surgery due to the advance diseases.

## MATERIALS AND METHODS

### Study population

We enrolled 214 patients with gastric cancer (GC) treated in our hospital from September 2004 to February 2008. These patients consisted of 146 male and 68 females. Median age at diagnosis was 66 years (ranged from 29 to 94 years). All GC was diagnosed histologically. Detailed information about histologic subtypes, anatomic location, macroscopic types, lymph node and other metastasis and peritoneal dissemination was also obtained according to the Japanese classification of gastric carcinoma [18]. Information about treatment of GC was available for 210 patients. 172 patients performed gastrectomy with consideration of surgical indication, while 36 patients were diagnosed as non-resectable and were received chemotherapy. Two patients received best supportive care due to advanced diseases without indication of any curative treatment. Overall survival, defined as the time from gastrectomy, or start of initial administration of chemotherapy to the date of cancer related death was determined for 210 patients. Recurrence free survival, defined as the time from gastrectomy, or start of initial administration of chemotherapy to the date of tumor progression or cancer related death was also determined in the same cases. Patients with no confirmation of progression or cancer related death were censored at the date of the last objective tumor assessment. The Ethics Committee of Fujita Health University School of Medicine approved the protocol and written informed consent was obtained from all of the subjects.

### Sample collection, assessment of H. pylori infection

During endoscopy, biopsy specimens were taken from primary GCs and adjacent normal-appearing mucosa. The specimens were immediately frozen and stored at −80°C until use. Genomic DNA was extracted directly from these frozen specimens using the standard protein precipitation method. All DNA samples were obtained before gastrectomy or chemotherapy. Through histological examination of biopsy specimens taken almost same area of the tumor tissue, we confirmed that all biopsies from cancerous tissue contained more than 70% of cancer cells. *H. pylori* infection status was assessed by histological analysis of both the biopsy specimens from the greater curvature of the gastric antrum and upper corpus using antibodies.

### Sanger sequencing

All coding regions of *TP53* gene (exon2 to exon11) from GC tissues and matched normal gastric tissues were amplified using PCR reactions. These PCR fragments were sequenced using Sanger Sequencing. PCR and sequence primers are listed in Supplementary Table 1. The sequence chromatograms were visually inspected with DNA Dynamo Sequence Analysis Software (Blue Tractor Software, Llanfair-fachan, Wales, UK). All mutations were confirmed by independent PCR and sequencing reactions. We only consider the nucleotide variation as the mutations, shown in the tumor samples, not in the normal tissues. For non-silent single nucleotide substitutions, SIFT (sorting intolerant from tolerant) analysis was performed to predict whether an amino acid substitution affects protein function.

### Statistics

The statistical significance of the differential frequency of *TP53* mutations in two groups was determined using two-tailed Fisher's exact test. Overall survival and recurrence free survival, in relation to the *TP53* mutations was assessed using the Kaplan-Meier method and the Log rank test. *P* value <0.05 was considered statistically significant.

## SUPPLEMENTARY MATERIALS




