# Supplementary material for: Mutation spectrum of TP53 gene predicts clinicopathological features and survival of gastric cancer
Source: Oncotarget. 2016 Jun 1;7(27):42252–60. doi: 10.18632/oncotarget.9770 (PMC5173132; doi:10.18632/oncotarget.9770)
Supplement: Supplementary file 1 [file oncotarget-07-42252-s001.pdf]

SUPPLEMENTARY MATERIALS

Supplementary Table 1: Primer assay used for sanger sequencing

Assay name	Forward primer sequence	Reverse primer sequence	Sequence primer sequence	PCR product size
Exon 2-4	tggaagtgtctcatgctgga	gccaggcattgaagtctcat	atgctggatccccacttttc	717bp
Exon 5-6	ccagttgctttatctgttcacttg	cactgacaaccacccttaacc	cacttgtgccctgactttca	496bp
Exon 7-8	aaaaggcctcccctgctt	aaaagtgaatctgaggcataactg	ccacaggtctccccaagg	730bp
Exon 9	cctttccttgcctctttcct	tgtctttgaggcatcatcactgc	ccccaattgcaggtaaaaca	226bp
Exon 10	acttctccccctcctctgtt	gaaggcaggatgagaatgga	gaatcctatggctttccaacc	219bp
Exon 11	aaagcattggtcagggaaaa	ggctgtcagtggggaaca	gggcacagaccctctcact	195bp

Supplementary Table 2: Summary of all 85 mutations detected in the dataset

See Supplementary File 1
